# Text Font Correction and Alignment Method for Scene Text Recognition

**DOI:** 10.3390/s24247917

**Published:** 2024-12-11

**Authors:** Liuxu Ding, Yuefeng Liu, Qiyan Zhao, Yunong Liu

**Affiliations:** School of Digital and Intelligent Industry, Inner Mongolia University of Science and Technology, Baotou 014010, China; 15524959095@163.com (L.D.); zhaoqiyan2022@163.com (Q.Z.); 13272042154@163.com (Y.L.)

**Keywords:** scene text recognition, text font alignment, feature fusion, attention

## Abstract

Text recognition is a rapidly evolving task with broad practical applications across multiple industries. However, due to the arbitrary-shape text arrangement, irregular text font, and unintended occlusion of font, this remains a challenging task. To handle images with arbitrary-shape text arrangement and irregular text font, we designed the Discriminative Standard Text Font (DSTF) and the Feature Alignment and Complementary Fusion (FACF). To address the unintended occlusion of font, we propose a Dual Attention Serial Module (DASM), which is integrated between residual modules to enhance the focus on text texture. These components improve text recognition by correcting irregular text and aligning it with the original feature extraction, thus complementing the overall recognition process. Additionally, to enhance the study of text recognition in natural scenes, we developed the VBC Chinese dataset under varying lighting conditions, including strong light, weak light, darkness, and other natural environments. Experimental results show that our method achieves competitive performance on the VBC dataset with an accuracy of 90.8% and an overall average accuracy of 93.8%.

## 1. Introduction

Scene Text Recognition (STR) is a long-standing computer vision task aiming to recognize the text from detected regions, as it has wide applications scenarios, such as text images on billboards, shop signs, and product labels, where font styles and curvatures differ significantly. The goal of STR is to extract text sequences from detected regions and cropped instances in real-world scenarios, accurately recognizing the text as correct characters. This area has garnered considerable attention, leading to a wide range of practical applications. Despite the significant progress [1,2,3,4,5,6,7,8] in scene text recognition in recent years, the variability in font styles and curvatures in natural scenes continues to hinder the accurate recognition of text.

**Figure 1:**
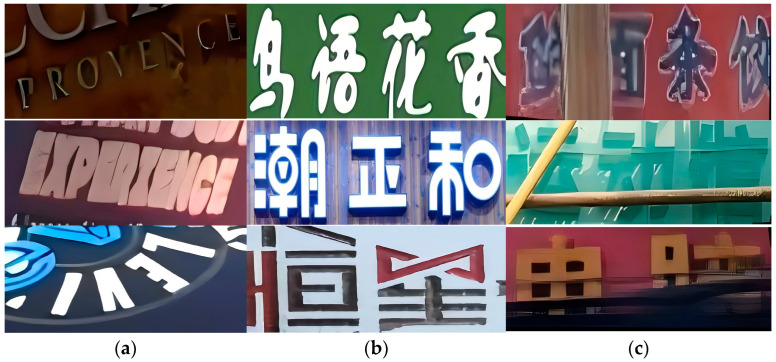
Illustrates various text images in natural scenes: (**a**) text images with different curvatures, (**b**) text images featuring different fonts, and (**c**) text images with occlusions. In panel (**b**), from top to bottom, the descriptions are “birdsong and fragrant flowers”, “proper season”, and “stars”. In panel (**c**), from top to bottom, the descriptions are “fresh noodles and dumplings”, “sports hall”, and “computer”.

**Figure 2:**
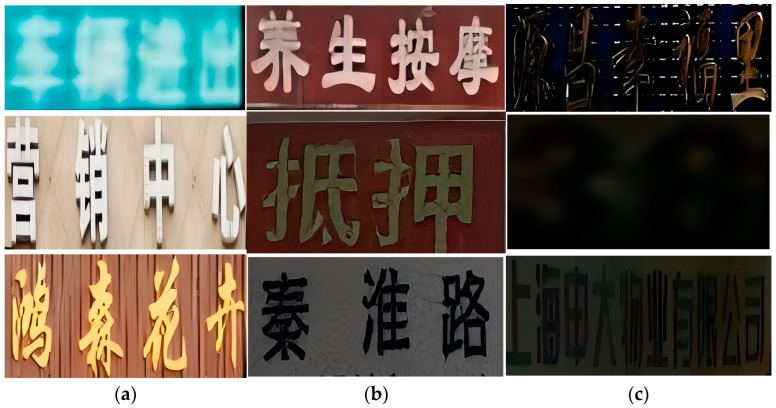
VBC dataset under varying lighting conditions. (**a**) images captured in natural daylight, (**b**) images captured under low light, and (**c**) images captured in complete darkness. In (**a**), from top to bottom, the descriptions are “vehicle entry and exit”, “marketing center”, and “Hongsen Floriculture”. In (**b**), from top to bottom, the descriptions are “health massage”, “mortgage”, and “Qinhuai Road”. In (**c**), from top to bottom, the descriptions are “Yuanchang Xingfuli”, “38”, and “Shanghai Shenda Property Co., Ltd”.

Despite recent advancements, scene text recognition still faces challenges related to font shapes, such as unintended font occlusion, irregular text arrangement, and uneven lighting conditions, as shown in Figure 1. Additionally, since most datasets are captured during the day, there is a lack of datasets for dimly lit and blurry scenes, resulting in poor performance of many recognition methods when applied to such scenes, as shown in Figure 2. To address these challenges, previous approaches have attempted to integrate related tasks into text recognition in these complex scenarios. Notably, some methods [9,10] combine Convolutional Neural Networks (CNNs) and Recurrent Neural Networks (RNNs) to enhance feature extraction and sequence modeling in text recognition, thereby improving the ability to extract text glyphs. For instance, the CRNN method [10] integrates CNN and RNN architectures, allowing for end-to-end training and the processing of text sequences of arbitrary lengths without the need for a predefined dictionary, significantly improving the recognition of similar text glyphs. Additionally, certain methods [11] leverage Generative Adversarial Networks (GANs) to improve text recognition, particularly in challenging scenarios. For example, Luo et al. [11] developed an attention-based recognizer combined with a GAN architecture that is capable of extracting text from irregular text glyphs, providing a novel approach to recognition.

Although significant progress has been made in text recognition, several challenges remain that necessitate further improvements in recognition methods to enhance accuracy. The first challenge is the arbitrary and random arrangement of text in natural scenes, such as on signs and billboards, which makes recognition more difficult. The second challenge involves the irregular and diverse font sizes and styles of text in images, which affect the accuracy of recognition methods. The third challenge stems from occlusion caused by variations in shooting angles and distances, which requires context-based recognition for accurate interpretation. These issues collectively lead to poor performance in such challenging scenarios.

To address these challenges, we propose the Text Font Correction and Alignment Method, which is a text recognition approach designed for varying natural lighting conditions. This method uses PlainMamba for feature extraction, which is processed by an FPN with a Dual Attention Serial Module (DASM) to obtain F, improving recognition performance. We also design a Discriminating Standard Text Font (DSTF) to effectively differentiate similar characters, enhancing feature recognition and text font identification. Additionally, we introduce the Feature Alignment and Complementary Fusion (FACF) module, which aligns and fuses the extracted features. FACF combines the features extracted by PlainMamba with those identified by DSTF, improving performance through efficient complementary fusion.

The main contributions of this paper are summarized as follows:

- We propose a Dual Attention Serial Module (DASM), which enhances the feature extraction of the FPN after PlainMamba with a minimal increase in model complexity, thereby contributing to improved recognition performance.
- We design the Discriminating Standard Text Font (DSTF) and Feature Alignment and Complementary Fusion (FACF) modules, which effectively correct irregular text. The corrected text features are then aligned and fused with the original network-extracted text features, thereby improving the model’s performance.
- We have established the VBC Chinese dataset, which includes natural scene images captured under different lighting conditions, such as dark and bright environments. This dataset provides valuable resources for researchers and promotes further research on text recognition in complex backgrounds.
- Our proposed method achieves a 2.2% improvement in accuracy over the existing state-of-the-art methods on the custom VBC dataset and a 2.0% improvement in average accuracy on public datasets, demonstrating the effectiveness of our recognition method. Furthermore, our dataset is publicly available.

## 2. Related Works

With the advancements in machine learning and deep learning, Scene Text Recognition (STR) has recently been regarded as a sequence generation task. During the encoding process for text recognition, STR models typically employ CNNs to encode images into feature spaces with the main differences among methods occurring in the decoding phase. Sequence generation based on the type of decoder can be categorized into two types: CTC-based methods and attention-based methods.

### 2.1. CTC-Based Methods

In text recognition, it is crucial for input and output to have a one-to-one correspondence and be well labeled. Connectionist Temporal Classification (CTC) addresses this alignment issue with a two-step decoding process: mapping character image subregions and applying CTC decoding [12]. CRNN [10] combines CNNs and RNNs to extract image features, addressing the sequence generation challenge. The CTC transcription layer predicts text sequences by removing duplicates and blank characters from the extracted feature maps. Rosetta [13] uses the CTC model in the text recognition phase to address the parameter count issue of the CHAR model. As a fully convolutional network, it can predict words of arbitrary lengths. GTC [14] leverages Graph Neural Networks (GCNs) and attention mechanisms to improve feature extraction and the CTC decoding process. PPOCR [9] uses simple and efficient CTC decoding to avoid misalignment between predictions and labels. SVTR [15] introduces a Transformer structure to more effectively extract information from text lines, achieving impressive results with CTC decoding alone. Overall, CTC resolves issues of sequence misalignment and text deformation during training while offering fast inference speeds.

### 2.2. Attention-Based Methods

**Figure 3:**
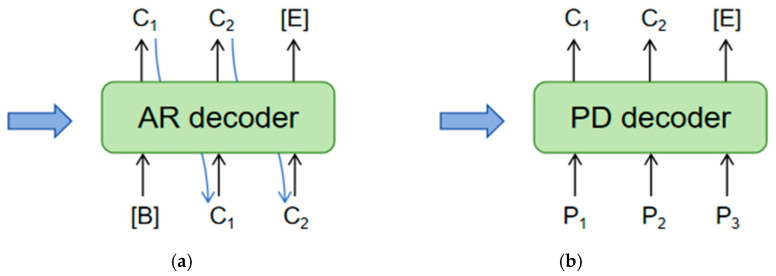
Illustrates the AR and PD decoders: (**a**) represents the Auto-Regressive (AR) decoder, while (**b**) depicts the Parallel Decoding (PD) decoder. The blue arrows indicate visual features.

Attention-based methods utilize Recurrent Neural Networks (RNNs) to interpret text semantics. These methods are divided into Auto-Regressive (AR) and Predictive Decoding (PD) models with specific differences in decoders illustrated in Figure 3. The AR decoder exhibits strong language modeling capabilities in text recognition and performs well in Natural Language Processing (NLP) tasks like machine translation [16,17,18]. However, its slower inference speed makes it less suitable for rapid text recognition tasks. In contrast, the PD decoder provides faster inference and can quickly predict individual characters. However, the absence of contextual relationships leads to lower accuracy compared to the AR decoder.

NRTR [19] developed an Auto-Regressive (AR) decoder using Transformer architecture for Scene Text Recognition (STR), achieving impressive results. Later, they incorporated ResNet as the encoder, further improving experimental performance. SATRN [20] uses a 2D Transformer to retain 2D features and employs self-attention mechanisms to capture spatial dependencies between characters in scene images. Xie et al. [21] developed a method for recognizing artistic text using an AR decoder to generate feature sequences, achieving notable accuracy. PARSeq [2] improves context capturing by using a permutation language model. This model learns a set of internally auto-regressive language models with shared weights, leading to enhanced accuracy. CDistNet [22] introduced the alignment of visual and semantic features through positional encoding. This approach addresses recognition difficulties caused by misaligned features from different domains and reduces attention drift, thereby improving text recognition performance.

SRN [23] handles a wide range of scene text by using multi-channel parallel propagation to capture global semantic information, enhancing Scene Text Recognition (STR) accuracy. CPPD [1] improves context modeling by incorporating character counting and sorting modules into the decoder, enhancing text recognition through ordered input. ABINet [24] uses bidirectional learning in the decoder to refine text representations, iteratively correcting predictions to reduce the impact of scene noise. However, this approach slows down inference speed. To handle occlusion in scenes, VisionLAN [25] introduces a Masked Language-Aware Module (MLM), which automatically generates mask mappings to guide character predictions. PIMNet [26] uses parallel attention for faster text prediction and an iterative generation mechanism to link contextual information, leading to more accurate predictions.

## 3. Methods

### 3.1. Overall Architecture

**Figure 4:**
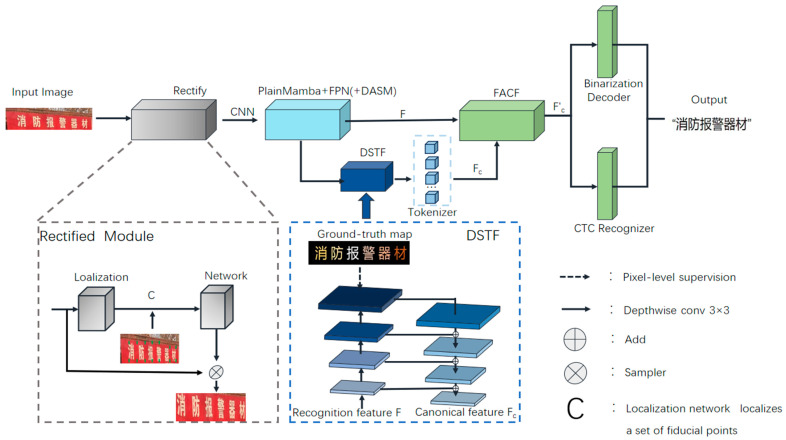
Illustrates the overall architecture of our proposed text font correction and alignment method. F represents the original features extracted by the backbone, while Fc denotes the masked features obtained after processing through the DSTF. $F_{c}^{\prime }$ represents the features obtained from the fusion and alignment of F and Fc in the FACF. In the overall architecture, the English meaning of the Chinese image is the fire alarm device.

The overall architecture of our Standard Text Font Alignment Recognition method is illustrated in Figure 4. The method begins with rectify processing to adjust the image text, followed by a CNN that flattens the features into a one-dimensional sequence, which is then input to PlainMamba. After sequence modeling with PlainMamba, the output is reshaped to map the sequence features back into a two-dimensional feature map. These reshaped features are then passed through the FPN with DASM for further feature extraction, resulting in the feature set F. The extracted features are subsequently processed by the Discriminative Standard Text Font (DSTF) module, forming the mask feature Fc. The original features F and the mask features Fc from the DSTF module are then aligned and fused using the Feature Alignment and Complementary Fusion (FACF) technique, leveraging their complementary information to enhance text recognition performance. Finally, the model outputs the recognition results through both the recognizer and binarization decoder, enabling efficient character sequence recognition and the accurate segmentation of character regions, thereby improving overall recognition accuracy. The rectify module employs a TPS-based STN, which is adapted from RARE [27].

### 3.2. Rectify

The rectification module employs a TPS-based Spatial Transformer Network (STN), as described in RARE [27]. The image rectification module employs a Spatial Transformer Network (STN) based on Thin-Plate Spline (TPS). By learning the TPS transformation parameters implicitly during recognition, this module adjusts text in scenes with non-standard layouts, boosting the accuracy of text recognition. This step supplies correctly adjusted inputs for the model’s subsequent operations, thereby enhancing overall performance.

### 3.3. Dual Attention Serial Module (DASM)

**Figure 5:**
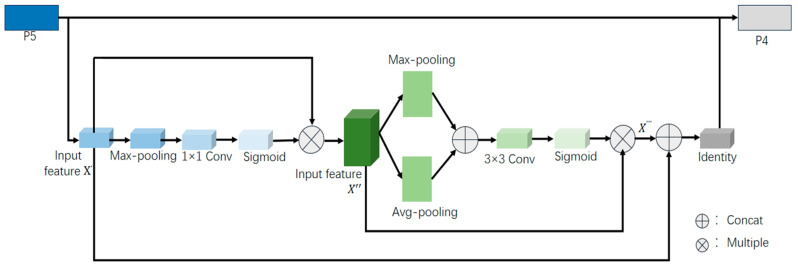
Illustrates the structure of the Dual Attention Series Module (DASM) integrated between each residual block.

**Figure 6:**
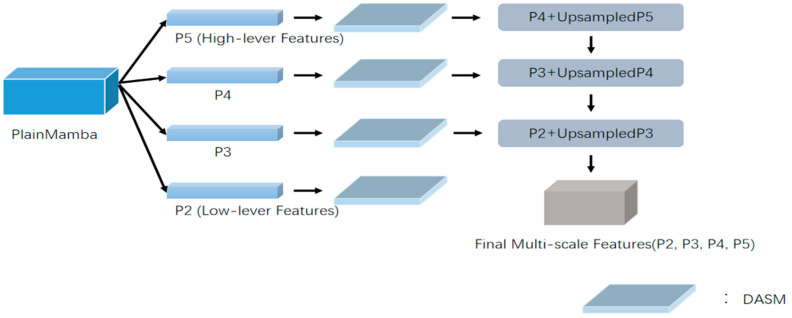
Overall structure diagram after inserting DASM into FPN.

We designed the Dual Attention Serial Module (DASM), which combines channel and spatial attention. Channel attention allows the network to focus on the features of image channels and assign corresponding weights, while spatial attention directs the network to concentrate on the text regions, ignoring irrelevant background details. The detailed structure of the DASM module, using P4 and P5 as examples, is illustrated in Figure 5. The DASM is inserted into the downsampling output stage of each FPN segment, as shown in Figure 6. 

For channel attention, we adopt the approach from [28], which adaptively recalibrates the weight information of each channel to emphasize the text regions in the image. Unlike [28], we replace the global average pooling layer with a global max pooling layer, tailored to the characteristics of our text dataset, to preserve more texture information for better recognition. Additionally, we substitute the fully connected layers in [28] with 1 × 1 convolutional layers, where the kernel size is determined by the number of input channels.

Specifically, based on F’, we map the input $X\in R^{H’\times W’\times C’}$ to the feature map $X^{\prime }\in R^{H\times W\times C}$. Here, F’ represents the convolution operator, and we denote the learned filter kernel set as $Y=\left[y_{1},y_{2},\ldots ,y_{c}\right]$, where $y_{c}$ represents the parameters of the c-th filter. We can express the output as $X^{\prime }=\left[x_{1}^{\prime },x_{2}^{\prime },\ldots ,x_{c}^{\prime }\right]$, as shown in the following equation:

(1) $$x_{c}^{\prime }=y_{c}*X=\sum_{n=1}^{C^{\prime }}y_{c}^{n}*x^{n}$$

where ∗ denotes the convolution operation. $Y_{c}=[y_{c}^{1},y_{c}^{2},\ldots ,y_{c}^{C^{\prime }}]$, $X=[x^{1},x^{2},\ldots ,x^{C^{\prime }}]$, $x_{c}^{\prime }\in R^{H\times W}$. $y_{c}^{n}$ is a two-dimensional spatial kernel, representing the effect of the $y_{c}$ filter on the corresponding channel of X.

However, each learned filter operates using a local receptive field, which means that each element of the output $X^{\prime }$ cannot utilize contextual information beyond that local area. Additionally, due to the cluttered backgrounds in scene images, it is crucial to retain more texture information. To address this issue, we propose introducing global maximum pooling into the channel descriptors. The statistic $w\in R^{c}$ is generated by compressing $X^{\prime }$ along its spatial dimensions H × W. The c-th element of w can be calculated as follows:

(2) $$w_{c}=W_{pq}(x_{c}^{\prime })=\underset{(p,q)\in R_{i,j}}{\max}x_{c}^{\prime }(p,q)$$

where $w_{c}$ represents the maximum pooling output value associated with the c-th element in the rectangular region $R_{i,j}$, where (p,q) denotes the position within the rectangular area $R_{i,j}$, and $x_{c}^{\prime }\left(p,q\right)$ is the element within that region. The output $X^{\prime }$ can be interpreted as a collection of local descriptors with the statistics being expressive for the entire image. Next, we opt to use a simple gating mechanism with a sigmoid activation, which is described by the following equation:

(3) $$m=F_{e}(w,S)=\sigma (g(w,S))=\sigma (S_{2}\alpha (S_{1}w))$$

where α represents the ReLU [29] activation function, $S_{1}\in R^{\frac{C}{r}\times C}$ and $S_{2}\in R^{C\times \frac{C}{r}}$. After passing through the 1×1 convolutional layer, the output is fed into the sigmoid activation function. Finally, the output after the sigmoid activation is multiplied by the input to obtain the final output, as described by the following equation:

(4) $$x_{c}^{\prime \prime }=F_{s}(x_{c}^{\prime },m_{c})=m_{c}x_{c}^{\prime }$$

where $X^{\prime \prime }=[x_{1}^{\prime \prime },x_{2}^{\prime \prime },\ldots ,x_{c}^{\prime \prime }]$ and $F_{s}(x_{c}^{\prime },m_{c})$ refers to the channel multiplication between the scalar $m_{c}$ and the feature map $x_{c}^{\prime }\in R^{H\times W}$. The features generated after the channel attention, denoted as $X^{\prime \prime }\in R^{H\times W\times C}$, are then input into the spatial attention module, where global maximum pooling $F_{max}\in R^{H\times W\times 1}$ and global average pooling $F_{avg}\in R^{H\times W\times 1}$ are performed along the channel dimension. The results from $F_{max}$ and $F_{avg}$ are concatenated, yielding a feature map of size $F_{co}\in R^{H\times W\times 2}$:

(5) $$F_{co}=concat\left(F_{max}{,F}_{avg}\right)$$

where a convolution operation is performed on the concatenated result, which is followed by inputting it into the sigmoid activation function to generate the spatial attention weights. The calculation formula is as follows:

(6) $$P=\sigma \left(f^{3\times 3}\left(F_{co}\right)\right)$$

where P is the resulting spatial attention weight matrix, and $f^{3\times 3}\left(\cdot \right)$ represents the output after the 3×3 convolution operation. Finally, the obtained P is multiplied by $X^{\prime \prime }$:

(7) $$X^{\prime \prime \prime }=PX^{\prime \prime }$$

where $X^{\prime \prime \prime }$ represents the result of multiplying the generated spatial attention weights P with the features obtained after the channel attention, which are denoted as $X^{\prime \prime }$.

### 3.4. Discriminating Standard Text Font (DSTF)

We use the Discriminating Standard Text Font (DSTF) module to enhance text recognition in cases of irregular text arrangement or inconsistent font shapes. The features F extracted during feature extraction are input into this module, which produces output features Fc with more discriminative visual information, improving recognition performance. Ultimately, we obtain the ground-truth map, where each pixel value represents the type of the corresponding character.

After rectifying and extracting features using PlainMamba, we obtain a feature map F, which is then input into a lightweight segmentation module with depth-wise convolution for pixel-level prediction. The composition is detailed in Table 1. The output prediction is $M\in R^{(N_{c}+1)\times H\times W}$, where $N_{c}$ represents the number of character classes with the remaining class corresponding to the background. In addition to predicting standard text font shapes, this module also feeds the generated features Fc along with the extracted features F into the next module to further enhance text recognition performance. As shown in the DSTF module in Figure 4, this component includes an upsampling decoder and a downsampling encoder, similar to the UNet [30] architecture, utilizing skip connections to better recognize text regions.

### 3.5. Feature Alignment and Complementary Fusion (FACF)

The features extracted in F and the features Fc obtained after the DSTF need to be aligned and fused to complement each other, thereby enhancing text recognition. However, we found that the mask alone cannot ensure pixel-level alignment between the feature maps and recognition features. To address this, we introduce a feature alignment and complementary fusion (FACF).

**Figure 7:**
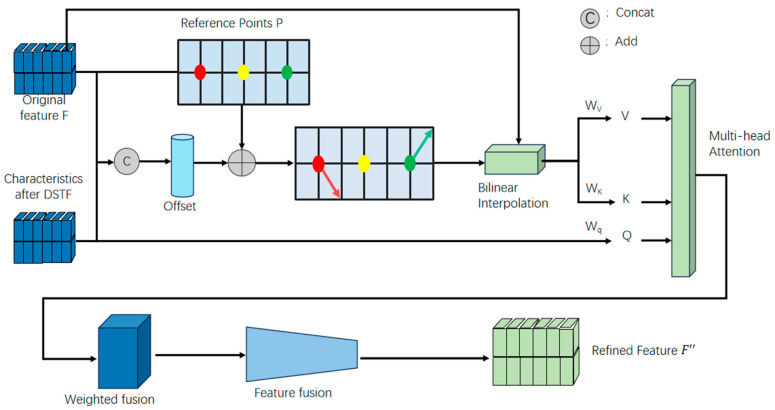
Shows the structure of the Feature Alignment and Complementary Fusion (FACF). In this module, a set of reference points is uniformly placed across the feature map, and the offset is learned by concatenating the recognition features F and the features Fc obtained from the Discriminating Standard Text Font (DSTF) module. This offset guides the alignment of the two feature sets. The reference points (red, yellow, and green) are evenly distributed across the recognition feature map, and the offsets are learned from the concatenated features of the recognition and normalized maps.

The structure of this module is illustrated in Figure 7. The original extracted features F and the features Fc from the DSTF pass a feature alignment process, where misaligned sampling points are adjusted until the features from both methods are properly aligned. Multi-head attention is then used to obtain corresponding weights for each part. Afterward, weighted fusion and feature merging operations are performed to generate the improved features $F_{c}^{\prime }$, which help enhance text recognition performance.

The features extracted in F and the features Fc obtained after the DSTF module are concatenated to facilitate the learning of the sampling point offsets. This enables the dynamic alignment of F and Fc:

(8) $$∆\theta =offset(concat\left(F,F_{c}\right))$$

where the offset required for feature alignment is represented. Then, the features Fo, which have undergone offset adjustment and bilinear interpolation, are used as the keys and values in the multi-head attention mechanism, while the features Fc obtained from the DSTF module are used as the query. The formula is as follows:

(9) $$F_{O}=\partial \left(F;\theta +∆\theta \right)$$

(10) $$Q=F_{c}Z_{q},K=F_{o}Z_{k},V=F_{o}Z_{v}$$

where ∂ represents bilinear interpolation, and $Z_{q}$, $Z_{k}$, and $Z_{v}$ denote the learnable parameters. Next, the query q, key k, and value v are input into the multi-head attention module. The computation for each attention head is as follows:

(11) $$F_{o}^{\prime }=softmax\left(\frac{q^{\left(d\right)}k^{(d)T}}{\sqrt{d}}\right)v^{\left(d\right)}$$

where $F_{o}^{\prime }$ represents the feature output from the multi-head attention, and d is the dimension of each attention head. Finally, after $F_{o}^{\prime }$ undergoes weighted fusion and feature merging, the improved feature $F_{c}^{\prime }$ is output as the final result.

### 3.6. CTC Recognizer and Binarization Decoder

In the final stage of text recognition, the enhanced features from Feature Alignment and Complementary Fusion (FACF) are fed into both the CTC recognizer and the binarization decoder. This parallel design leverages the CTC recognizer’s global sequence prediction capabilities while integrating the pattern detection strength of the binarization decoder to better handle local features and irregular fonts, improving recognition efficiency and robustness.

Although the CTC recognizer excels at global sequence modeling, it struggles with expressing local features and refining symbol detection. To address this, the binarization decoder focuses on specific local regions, complementing the CTC’s capabilities in local feature modeling. This combined approach enhances recognition, particularly for arbitrary text arrangements, irregular fonts, and occlusions. The detailed experimental results in Table 2 demonstrate the effectiveness of incorporating the binarization decoder.

### 3.7. Loss Function

The overall loss function consists of two components: the enhanced Connectionist Temporal Classification (CTC) loss and the pixel-level segmentation loss.

(12) $$L_{total}=L_{ectc}+{\alpha L}_{seg}$$

where α is set to 1.0 through empirical experimentation.

Enhanced CTC Loss. CRNN [10] serves as the fundamental architecture for text recognition, integrating feature extraction and sequence modeling. It uses the Connectionist Temporal Classification (CTC) loss to avoid inconsistencies between the predicted results and the ground truth. Since the network is trained from scratch, the CTC loss is applied during training, and the formula is as follows:

(13) $$L_{ctc}=CTC\left(H_{s},gt\right)$$

where Hs represents the network’s head output, and gt denotes the ground-truth labels of the input image. Additionally, $H_{s}$ can be calculated as follows:

(14) $$H_{s}=Softmax(H_{s})$$

In Chinese recognition tasks, there is a phenomenon of glyph similarity, where characters with minor visual differences can be easily misrecognized. To address this, we incorporate CenterLoss [31], adapting it to the sequence recognition task. The definition is as follows:

(15) $$L_{ectc}=L_{ctc}+\beta L_{center}$$

where β is set to 0.05. The $L_{center}$ formula is expressed as follows:

(16) $$L_{center}=\sum_{i=1}^{I}{\left\|r_{i}-C_{s_{i}}\right\|}_{2}^{2}$$

where $r_{i}$ represents the feature at timestamp t, and $C_{s_{i}}$ is the center for class $s_{i}$. Since in CRNN [10] the features and labels are misaligned, the explicit label $s_{i}$ for $r_{i}$ cannot be directly obtained. To acquire $s_{i}$, we employ a greedy decoding strategy, and the formula is as follows:

(17) $$s_{i}=argmax\left(q\times r_{i}\right)$$

where q represents the parameters of the CTC head.

Pixel-Level Segmentation Loss. We use the cross-entropy loss for training, and the prediction loss is defined as follows:

(18) $$L_{seg}=-\frac{1}{M\times N}\sum_{m=1}^{M}\sum_{n=1}^{N}O_{m,n}\left(\sum_{i=0}^{H_{i}}1\left(W_{m,n}^{\prime }==i\right)\log\left(\frac{e^{W_{i,m,n}}}{\sum_{k=0}^{H_{i}}e^{W_{i,m,n}}}\right)\right)$$

where $W_{i,m,n}$ represents the elements of the predicted map, and $W_{m,n}^{\prime }$ denotes the corresponding class label. $m=\left\{1,2,\ldots ,M\right\}$, $n=\left\{1,2,\ldots ,N\right\}$ and $i=\left\{1,2,\ldots ,H_{i}\right\}$. The function $1\left(\cdot \right)$ is an indicator function, and $O_{m,n}$ represents the corresponding weight for each pixel. The weight calculation is as follows:

(19) $$O_{m,n}=\left\{\begin{array}{c} \frac{U_{n}}{U-U_{n}},\;\;\;if\;W_{m,n}^{\prime }>0\\ 1,\;\;\;\;\;\;\;\;otherwise \end{array}\right.$$

where U=M×N represents the total number of pixels, and $U_{n}$ denotes the number of background pixels.

## 4. Experiments

### 4.1. Datasets

The text recognition method proposed in this paper was evaluated on multiple datasets to ensure its effectiveness and reliability in both Chinese and English scene text recognition. The Chinese datasets include our custom VBC dataset and the public CTR dataset [32]. The English datasets consist of the regular ICDAR2013 [33] and Street View Text (SVT) [34] datasets as well as the irregular ICDAR2015 [35], Street View Text-Perspective (SVTP) [36], and COCO-text [37] datasets. Additionally, we conducted training and testing on several multilingual and bilingual datasets, including the IC2017-MLT [38], IC2019-ArT [39], CTW1500 [40], and MSRA-TD500 [41] datasets.

#### 4.1.1. Chinese Datasets

VBC Dataset: This dataset includes images captured using cameras, mobile phones, and a smaller portion from watermarked images found in online media. It consists of 3000 samples with 2400 images used for training and 600 images used for testing.

CTR Dataset [32]: This dataset is divided into four parts: Scene, Web, Document, and Handwriting. The Scene part provides 509,164 samples for training and 63,645 samples for testing. The Web part contains 20,000 text images from 17 different categories on the Taobao website.

#### 4.1.2. Regular English Scene Datasets

ICDAR2013 Dataset [33]: This dataset uses the top-left and bottom-right points for annotation. It includes 229 English scene images for training and 233 English scene images for testing.

SVT [34]: This dataset contains 350 text images that capture the variability of real-world scenes. The SVT dataset presents diverse challenges regarding resolution and scene complexity, making it an indispensable resource for evaluating text recognition models. Its varied conditions allow for a comprehensive assessment of a model’s ability to handle different resolutions and complexities in real-world environments.

#### 4.1.3. Irregular English Scene Datasets

ICDAR2015 [35] utilizes a four-point annotation format, comprising a training set of 1000 English scene text images and a testing set of 500 images. This annotation method not only enhances the accuracy of text region localization but also provides rich and detailed samples for model training and evaluation, contributing to improved text recognition performance.

The SVTP [36] dataset includes 238 images, which correspond directly to those in the SVT [34] test set. However, the shooting angles of the images in SVTP [36] differ from those in SVT [34], introducing additional challenges for recognition.

The COCO-text dataset [37] is derived from the popular COCO dataset and contains 22,184 training images and 7026 validation images. It features images from various real-world scenarios, including complex backgrounds and crowded scenes, making it a valuable resource for scene text recognition.

#### 4.1.4. Multilingual Scene Text Dataset

The IC2017-MLT dataset [38] is a comprehensive multilingual scene text dataset designed for text detection and recognition. It includes 12 languages, such as English, Arabic, and Chinese, with 7200 annotated images for training and testing. The dataset presents challenging real-world scenes, featuring complex layouts, diverse fonts, and multiple languages, making it highly valuable for multilingual text recognition tasks.

#### 4.1.5. Chinese and English Complex Scene Datasets

The IC2019-ArT dataset [39] focuses on artistic text recognition and includes 5603 training images and 4573 test images. The dataset contains graffiti, murals, and stylized fonts, presenting challenges such as diverse artistic styles, font distortions, and occlusions for text recognition models.

The CTW1500 dataset [40] is designed for text recognition in curved and irregular layouts. It consists of 1000 training images and 500 test images, capturing various text shapes and orientations in complex scene settings. This dataset is ideal for evaluating models that handle curved text and distorted characters in real images.

The MSRA-TD500 dataset [41] contains 500 high-resolution images featuring a variety of text types, including natural scene text, documents, and signage. This dataset is suitable for testing model generalization, as it includes both Chinese and English scenes, making it ideal for evaluating the performance of text recognition models in real-world conditions.

### 4.2. Implementation Details

We used the Adam optimizer to train the recognition model, employing a stepwise decay strategy with an initial learning rate of 0.001. At the beginning of training, we performed five warm-up epochs specifically for the recognition model, which helped improve text recognition performance. During training, we applied common data augmentation techniques, such as random rotation, perspective distortion, motion blur, and Gaussian noise, to enhance the robustness and generalization ability of our proposed model.

Since most recognition tasks involve scene text of moderate length, we set the maximum predicted text length for most images to 25. However, for the CTR dataset, which includes the Scene, Web, Document, and Handwriting categories, we adjust the maximum predicted text length to 40.

Additionally, to ensure clarity in pixel-level segmentation, our architecture makes text case insensitive, eliminating any pixel-level semantic ambiguity, which benefits English text recognition. The evaluation metrics for both Chinese and English text recognition are accuracy and average accuracy.

### 4.3. Comparison with Other Benchmark Dataset

#### 4.3.1. Differences Between the Custom VBC Dataset and Public Datasets

The model we proposed was evaluated on both English and Chinese datasets. For the public English datasets, we primarily used the regular ICDAR2013 [33] and Street View Text (SVT) [34], as well as the irregular ICDAR2015 [35], Street View Text-Perspective (SVTP) [36], and COCO-text [37]. For Chinese datasets, we used the CTR dataset [32]. Additionally, we evaluated our model on bilingual datasets such as IC2019-ArT [39], CTW1500 [40], MSRA-TD500 [41], and the multilingual IC2017-MLT [38]. A comparison between these public datasets and our proposed VBC dataset is provided, highlighting differences in test images, types, and languages, as shown in the Table 3.

Moreover, as described in Section 4.1, only the CTR [32], COCO-text [37], IC2017-MLT [38], and IC2019-ArT [39] datasets contain annotated text lines. In contrast, the remaining public datasets and our custom VBC dataset consist of images that have not been segmented into text lines. This distinction is important to note. The “Test images” mentioned in this section refer to images that have been segmented into text lines.

**Figure 8:**
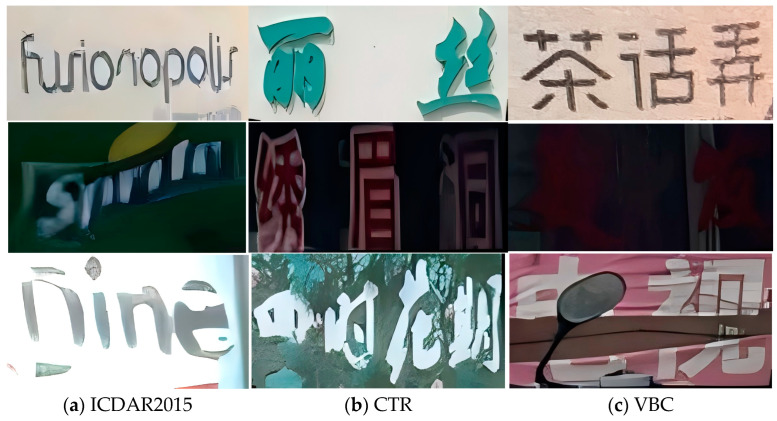
Highlights the differences between the custom VBC dataset and public datasets. In panels (**a**–**c**), the scenarios represent daytime, nighttime, and occlusion conditions, respectively, from top to bottom [32,35]. In panel (**b**), from top to bottom, the Chinese translations are “Lis”, “Xiumei Cave”, and “Four Seasons Flowering Period”. In panel (**c**), from top to bottom, the Chinese translations are “Teahouse Alley”, “Hairdressing”, and “Television”.

To visually compare the differences between the custom VBC dataset and the public datasets, we randomly selected text images from the VBC dataset, ICDAR2015 [33], and CTR [32]. As shown in Figure 8, our custom VBC dataset is more challenging to recognize in nighttime and occlusion scenarios compared to other public datasets.

#### 4.3.2. Accuracy of the Custom VBC Dataset vs. Public Datasets

Following the existing Scene Text Recognition (STR) framework, we systematically compare the proposed method with the latest state-of-the-art (SOTA) methods, which perform exceptionally well on four commonly used benchmark datasets. To further validate the effectiveness and accuracy of our method under different lighting conditions, we also conducted experiments on the custom VBC dataset, which includes a wide range of complex scenes with varying lighting conditions. We ran multiple SOTA methods under identical hardware conditions with accuracy as the consistent evaluation metric to ensure a fair comparison. The specific experimental results are presented in Table 4, showing that our proposed architecture outperforms existing methods across models of various scales, particularly when handling complex scenes and diverse data. Additionally, Table 5 provides the parameters (×10^6^) and FPS (Frames Per Second) metrics for the public datasets, demonstrating that our method enhances performance without compromising model efficiency.

From the data in Table 4, it is evident that our method achieves impressive accuracy, reflecting its superior performance. Compared to the recent LPV-B [4] method, our approach shows an average accuracy improvement of 2.0% across multiple benchmark tests, highlighting its robustness and effectiveness.

**Figure 9:**
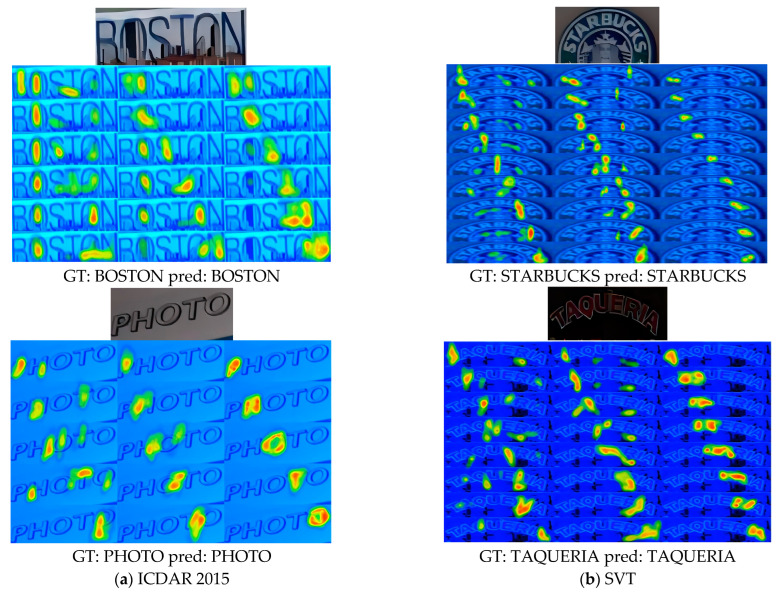
Visualization of attention heatmaps. For each example, the heatmaps, from left to right, represent the baseline model, the attention heatmap using a ResNet-34 encoder, and the attention heatmap using a PlainMamba encoder. Here, “GT” represents the ground truth, “pred” represents the prediction from our model, and the top left corner shows the original image.

**Figure 10:**
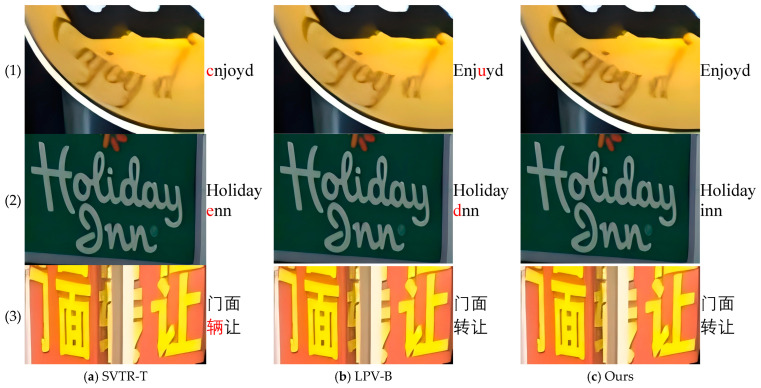
Visualization of text recognition results in three challenging scenarios: arbitrary text arrangements, irregular text shapes, and unexpected font occlusions. Specifically, (1) illustrates arbitrary text arrangements in the ICDAR 2015 dataset, (2) depicts irregular text shapes in the SVT dataset, and (3) shows unexpected font occlusions in the custom VBC dataset. For each image, the text region displays the recognition results, with (**a**,**b**) showing the results compared to other models, and (**c**) presenting the results of our proposed model. Red highlights indicate incorrectly recognized parts, while black highlights show correctly recognized parts [4,43]. The column in (3) is in Chinese, with the English translation as “storefront transfer”.

Furthermore, Figure 9 and Figure 10 demonstrate the recognition performance of our proposed model in three challenging scenarios: arbitrary text arrangement, irregular text glyphs, and unexpected font occlusions. These results are compared with visualizations from other models, further highlighting the efficiency and accuracy of our model in handling complex real-world application scenarios. Specifically, in Figure 9, we present the recognition results and attention heatmaps of our model on different datasets to visually validate its effectiveness. In particular, we selected two challenging text instances from the ICDAR2015 and SVT English datasets that align with the scenarios addressed by our model, such as arbitrary text arrangement, irregular text glyphs, and unexpected font occlusions. Through the visualization analysis of the baseline model and different encoders on these two public datasets, we aim to more intuitively demonstrate that changing the encoder is effective, highlighting the advantages of our final model in text recognition tasks. Additionally, in Figure 10, we show the visual recognition results of SVTR-T [43], LPV-B [4], and our model on these challenging text instances.

#### 4.3.3. CER and WER Metrics on Public Datasets

To further evaluate the effectiveness of our model, we assessed its performance on public datasets using two widely-used metrics for scene text recognition: Character Error Rate (CER) and Word Error Rate (WER). These error metrics provide a meaningful measure of the model’s performance. We performed inference with the trained model and compared the predicted results to the ground truth labels in order to calculate the CER and WER values. Table 6 presents the quantitative results of our method, comparing it with existing methods on five commonly used complex public datasets. It is clear that our method consistently outperforms the others across several datasets.

#### 4.3.4. Efficiency Comparison Across Different Models

To thoroughly evaluate our model’s performance, we utilize three key metrics: Accuracy, FPS (Frames Per Second), and Parameter Count. These metrics assess the model’s performance, efficiency, and complexity, providing a comprehensive comparison against other methods to highlight the advantages of our approach. The specific values for these metrics are presented in Table 5.

Accuracy directly reflects the model’s performance, evaluating its output and predictive capabilities. FPS (Frames Per Second) measures the model’s computational efficiency, indicating the speed of inference and associated time complexity. Parameter Count indicates the model’s scale and complexity, which are typically correlated with the number of layers and neurons. A higher parameter count generally suggests a more complex model.

**Figure 11:**
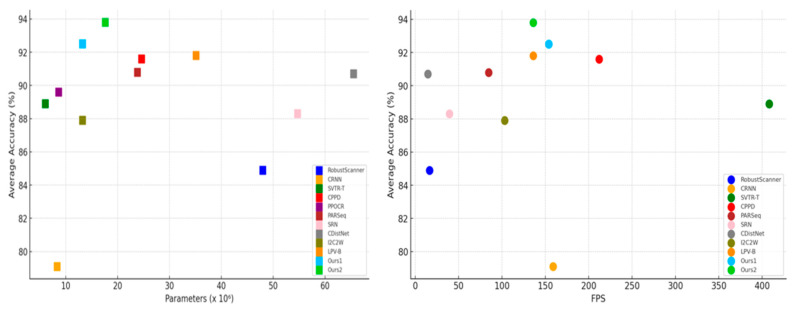
Comparison of Average Accuracy (%), Parameter Count, and FPS of different models across datasets.

From Table 5, it is evident that our method excels in Accuracy, FPS, and Parameter Count. Specifically, it maintains the highest Average Accuracy (%) while keeping the parameter count efficient and achieving a high FPS. This balance makes the model well suited for resource-constrained devices, ensuring excellent performance without compromising computational efficiency. Additionally, the high FPS supports real-time processing, making the model ideal for applications that demand low latency. To further highlight the effectiveness of our method, Figure 11 compares the Average Accuracy (%), Parameter Count, and FPS across various models on the VBC, ICDAR 2013, SVT, ICDAR 2015, and SVTP datasets, offering a clear, visual representation of the advantages of our approach.

#### 4.3.5. Recognition on CTR Chinese Dataset

The CTR Chinese dataset comprises real-world Chinese scene images, reflecting a diversity of data. To further validate the effectiveness and generalization ability of our proposed method, we conducted experiments on the CTR Chinese dataset, which includes four categories: Scene, Web, Document, and Handwriting. The specific results are presented in Table 7. These findings further confirm the effectiveness of our method. Under the same experimental settings, our proposed architecture achieved state-of-the-art average performance on the CTR dataset. Compared with the CPPD [1] model, our method improved the average performance by 0.8%, demonstrating its effectiveness.

### 4.4. Comparison of Different Encoders

The traditional ResNet framework addresses the gradient vanishing problem in deep networks by utilizing residual connections, ensuring stable performance during training. SVTR-Base is a variant of the Swin Transformer, which is specifically tailored for text recognition. It reduces the computational cost of global attention using the window attention mechanism while maintaining strong local feature extraction capabilities and global dependency modeling, making it highly effective for scene text recognition. PlainMamba, derived from the sequence modeling framework Mamba, is adapted for vision tasks. It achieves linear time complexity through the selective state space module (SSM), making it particularly suitable for handling complex dependencies and resource-constrained environments.

In this study, we evaluate three encoders for text recognition: the traditional ResNet, the SVTR-Base variant of the Swin Transformer, and PlainMamba, which is the vision-oriented extension of Mamba. All models will be trained and tested on our custom VBC Chinese scene dataset. The evaluation metrics include Accuracy, FPS, and Parameter Count, which comprehensively reflect the impact of these encoders on model performance and efficiency. The results are summarized in Table 7 below.

From the comparison in Table 8, it is evident that PlainMamba as the encoder achieves the highest accuracy, reaching 90.8%, outperforming the other two encoders. In terms of model efficiency, PlainMamba maintains a balanced Parameter Count and FPS, demonstrating a good trade-off between performance and computational efficiency. Overall, using PlainMamba as the encoder strikes an optimal balance, making it well suited for complex text recognition tasks that require both high accuracy and resource efficiency.

### 4.5. VBC Dataset Under Different Lighting Conditions

The VBC dataset primarily includes natural environments with varying lighting conditions, such as strong light, weak light, and darkness. We divided the dataset into four main scenes: strong light, weak light, lamplight, and darkness. The fully trained model was tested on these four scenes to assess differences in recognition accuracy under different lighting conditions. Table 9 shows the test text line images and their corresponding accuracy for each scene.

As shown in Table 9, there are indeed differences in recognition accuracy across lighting conditions. Specifically, the lowest accuracy occurs in the dark scene, while the highest is observed under lamplight. This is because the lamplight scene typically has evenly distributed light, resulting in high contrast between the text and background, which helps the model better capture text edge features, leading to the best performance. In contrast, low-light conditions often require higher ISO sensitivity, which increases image noise and blurs text edges, reducing the brightness difference between the text and background. This results in a significant reduction in contrast and, consequently, the lowest recognition accuracy in dark environments.

### 4.6. Comparison of Chinese-English Mixed Text Public Datasets

To further validate the excellent generalization performance of our model, we plan to compare it with various text recognition models using three datasets: IC2019-ArT, CTW1500, and MSRA-TD500. As shown in Table 10, our method outperforms other models across all evaluation metrics for each dataset, achieving the best results. Specifically, the average accuracy is improved by 3.3% compared with state-of-the-art (SOTA) models. This demonstrates that our model not only possesses strong recognition capabilities for single languages but also exhibits robust generalization in complex scenes and bilingual scenarios, such as Chinese–English mixed environments, thereby adapting effectively to real-world applications.

### 4.7. Ablation Study

In this section, we primarily analyze three components: the Dual Attention Serial Module (DASM), Discriminative Standard Text Font (DSTF), and Feature Alignment and Complementary Fusion (FACF). Together, these components form the recognizer used for text recognition. We evaluated the contribution of each module to the overall recognition performance on our self-built VBC dataset, ICDAR2015 [35], and SVTP [36], demonstrating the effectiveness of each module. The results are shown in Table 11, where the Discriminative Standard Text Font (DSTF) and the Feature Alignment and Complementary Fusion (FACF) complement each other, working synergistically to enhance recognition performance. Therefore, in the ablation experiments, both modules were combined for evaluation.

The experimental results in Table 11 indicate that introducing the Dual Attention Serial Module (DASM) improved recognition performance by 0.9%, 0.4%, and 0.7% on the VBC, ICDAR 2015 [35], and SVTP [36] datasets, respectively. This suggests that DASM significantly enhances performance in complex scenarios across various datasets. Furthermore, replacing the ResNet encoder with PlainMamba resulted in a more substantial improvement with increases of 1.3%, 2.1%, and 1.6% on the VBC, ICDAR 2015, and SVTP datasets, respectively. Additionally, when PlainMamba, DASM, the Discriminative Standard Text Font (DSTF) module, and the Feature Alignment and Complementary Fusion (FACF) module were combined, recognition performance further improved with gains of 2.4% on the VBC dataset and 2.5% and 2.6% on the ICDAR 2015 and SVTP datasets, respectively. These results demonstrate that the synergistic effect of DASM and the FACF module significantly enhances recognition tasks across various datasets.

**Figure 12:**
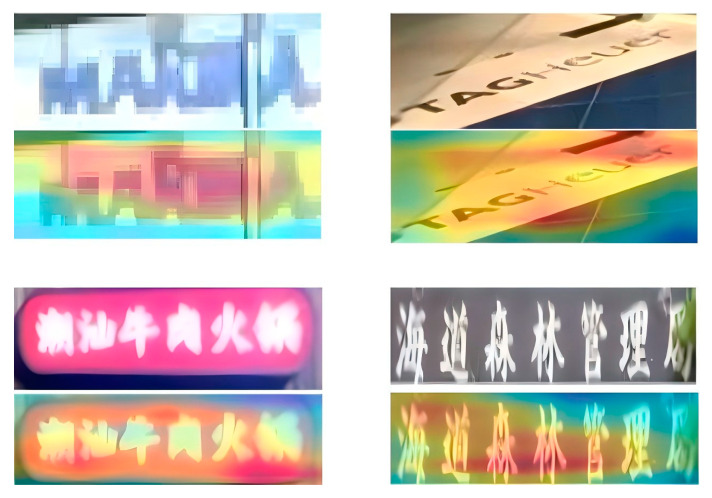
Visualization of text regions after encoding. For each example, the original image is displayed on top, and the heatmap generated by the PlainMamba encoder is shown below it. The two images below are in Chinese, with the English translations, from left to right, as “Chaozhou Beef Hotpot” and “Haidao Forest Management Bureau”.

We plan to generate heatmaps after the PlainMamba encoder to display the attended text regions more intuitively, thus demonstrating that the design of the model’s encoder is both reasonable and effective. As shown in Figure 12, the heatmap generated after the encoder clearly highlights the attended text regions, with red areas concentrated on the text, confirming that our PlainMamba encoder is indeed effective.

**Figure 13:**
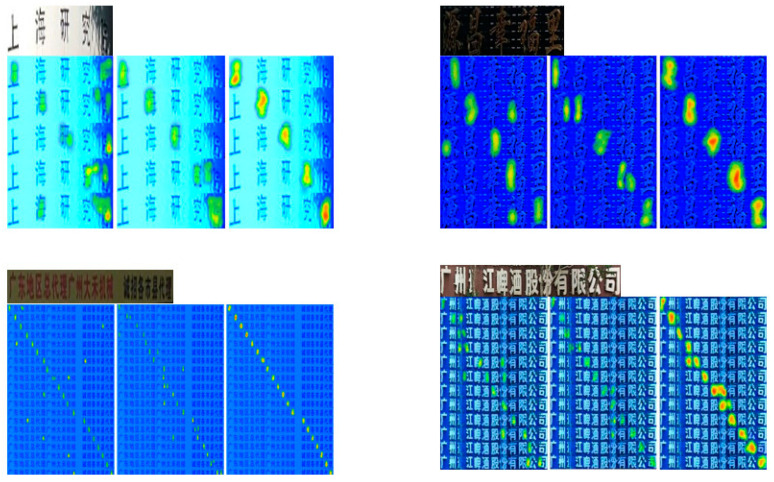
Visualization of the results after ablating each module on the VBC dataset. For each example, the text instances obtained using the Text Font Correction and Alignment Method, along with their recognition results, are presented. From left to right, the three columns show the visualizations of the recognition features: using PlainMamba with DASM, using DSTF and FACF, and the final recognition features. Since VBC is a Chinese dataset, the attention heatmaps displayed are all in Chinese. In the first row, from left to right, the English translations are “Shanghai Research Institute” and “Yuanchang Xifuli”. In the second row, from left to right, the English translations are "Guangdong Regional General Agent, Guangzhou Dahe Machinery is recruiting agents in various cities and counties" and "Guangzhou Jiang Beer Co., Ltd”.

**Figure 14:**
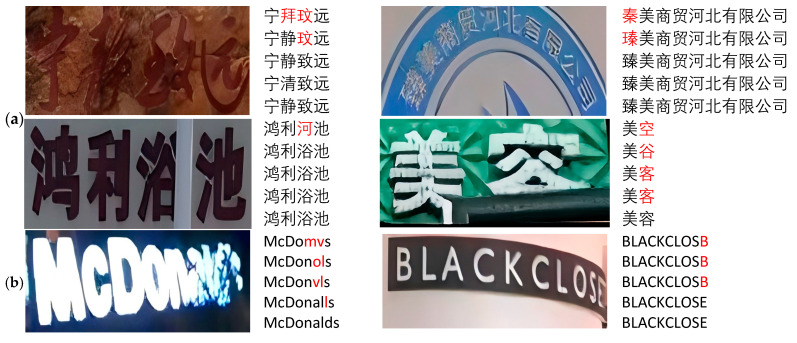

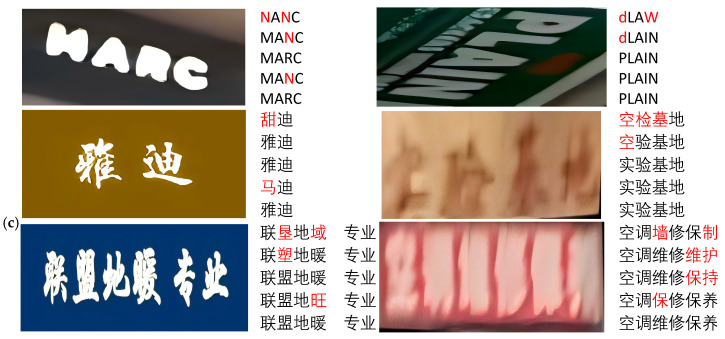
Visualization of text recognition results, where (**a**–**c**) represent three different datasets, demonstrating the usefulness of each module in our method. The text areas next to each image, from top to bottom, represent the baseline model, the addition of DASM, the replacement of the encoder with PlainMamba, the combination of the DSTF and FACF modules, and the overall recognition performance of the model architecture. Red regions indicate recognition errors, while black regions represent correctly identified areas. The images in (**a**,**c**) contain Chinese text. In (**a**), the English meanings of the first row, from left to right, are “Serenity Leads to Greatness” and “Zhenmei Trading Hebei Co., Ltd”. The second row translates to “Hongli Bathhouse” and “Beauty Salon”. In (**c**), the English meanings of the first row, from left to right, are “Yadi” and “Experimental Base”. The second row translates to “Alliance Underfloor Heating Specialist” and “Air Conditioning Maintenance and Repair”.

To provide a more intuitive demonstration of how each module in our model addresses the three challenges, we present visualizations of text recognition on the VBC dataset, as shown in Figure 13. These attention heatmaps clearly illustrate the differences in how various methods handle text images, further validating the effectiveness of our approach. Specifically, the three columns, from left to right, represent the visualizations of features obtained by adding PlainMamba and DASM, DSTF and FACF, and the final set of recognized features, respectively. Additionally, the final recognition results produced by these modules are displayed sequentially from top to bottom, as shown in Figure 14.

### 4.8. Visual Text Font Masks

**Figure 15:**
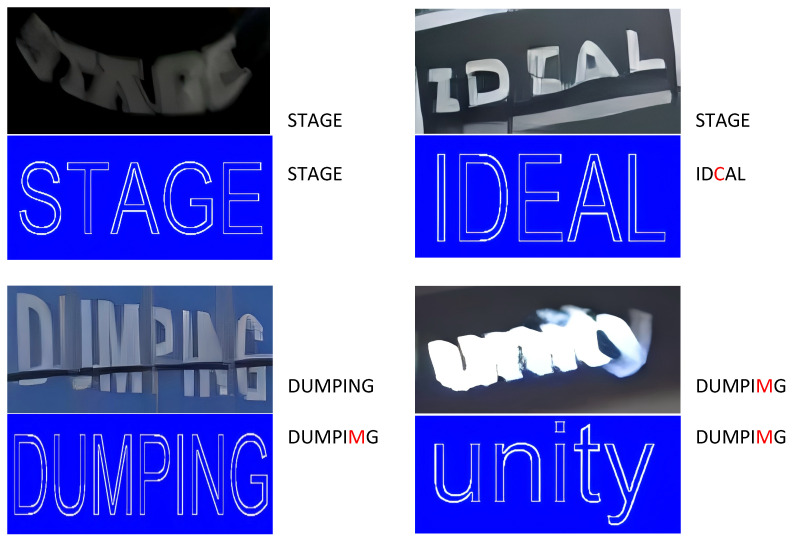
Shows the visualization of the text font masks generated by the model without refinement. The text region on the right side of the image displays the ground truth at the top and the predicted results at the bottom.

The DSTF module helps improve text recognition in challenging scenarios. Although some text recognition results at this stage are not fully accurate, the module effectively predicts and aligns the normalized text mask in particularly difficult situations. The mask demonstrates its effectiveness in aligning irregular text with a standardized layout, adjusting text angles, and distinguishing different fonts. Figure 15 shows the visualization after applying the Discriminative Standard Text Font (DSTF), illustrating its impact on enhancing text alignment and recognition in complex scenes.

### 4.9. Ablation Experiments of Recognizer and Binarization Decoder

In this study, part of the performance gains can be attributed to the effective use of the Feature Alignment and Complementary Fusion (FACF). To more comprehensively and efficiently distinguish similar fonts and enhance text recognition performance, we conducted a series of experiments involving various font types, including different English and Chinese fonts, for further assessment.

In the Scene Text Recognition (STR) task, the model utilizes both a CTC recognizer and a binarization decoder, enabling it to effectively recognize character sequences while precisely segmenting character regions. This dual-strategy approach combines the efficient sequence decoding capabilities of the CTC recognizer with the precise segmentation functionality of the binarization decoder, significantly improving the model’s overall performance.

As shown in the experimental results in Table 11, while different datasets may have a slight impact on final recognition performance, the joint use of the recognizer and binarization decoder consistently enhances recognition outcomes across various scenarios. Compared to using either the recognizer or other decoding methods alone, the synergy between the two modules demonstrates clear advantages in improving both recognition accuracy and segmentation precision. This confirms the reliability and applicability of the combined approach in complex environments.

## 5. Conclusions

To develop an effective model for challenging scene text recognition, we created the VBC dataset, which features complex backgrounds, varying lighting conditions, and blurred scene text. We compared recent methods on this dataset, and the results indicate that our proposed method achieved impressive performance. Specifically, our method introduces three main components: the Dual Attention Serial Module (DASM), the Discriminative Standard Text Font (DSTF) module, and the Feature Alignment and Complementary Fusion (FACF) module. The DASM module better distinguishes background from non-text regions, while the DSTF module corrects curved text, enabling the more accurate recognition of the processed text images. The FACF module aligns the original feature extraction with the features processed by the DSTF module, leveraging complementary information to help distinguish visually similar characters.

We visualized the recognition results from the three main modules, demonstrating that each module is effective. We also tested our proposed method on public datasets to validate its robustness and generalization ability, showing varying degrees of accuracy improvement. Therefore, future research will focus on exploring effective text recognition methods to address current limitations in recognizing incorrect text. Additionally, we are interested in integrating our proposed method with recent large language models [56,57], which have shown impressive capabilities in text detection and recognition tasks. Effectively combining these approaches is a topic worth further investigation.

## Figures and Tables

**Table 1:** Structure of the lightweight segmentation module with depth-wise convolution. The configuration outlines the components at the current stage: DepthConv represents depth-wise convolution, BN denotes batch normalization, and FC refers to the fully connected layer.

Layer	Configuration
Stage 1	DepthConv
	BN
Stage 2, Stage 3 and Stage 4	Upsample
	DepthConv
	BN
	ReLU
	DepthConv
	BN
FC	-

**Table 2:** This section presents experiments using different datasets to validate the feasibility of both the theoretical and practical approaches for the recognizer and binarization decoder. The bolded numbers indicate better performance on these datasets.

CTC Recognizer	Binarization Decoder	VBC	ICDAR2013	SVT	ICDAR2015	SVTP	Average Accuracy (%)
✔		89.9	97.5	95.8	88.2	92.8	92.8
✔	✔	**90.8**	**98.3**	**97.1**	**89.5**	**93.4**	**93.8**

**Table 3:** Comparison of dataset differences in scene text recognition.

Benchmarks	Test Images	Description	Language	Light Condition
ICDAR2013 [33]	1015	Regular Scene Text	English	Daytime and standard lighting conditions
SVT [34]	647	Regular Scene Text	English	Daytime and standard lighting conditions
ICDAR2015 [35]	1811	Irregular scene text	English	Daytime and standard lighting conditions
SVTP [36]	645	Irregular scene text	English	Variations in lighting, including sunlight and shadows
CTR [32] Scene	63,645	Irregular scene text	Chinese	Natural sunlight, indoor lighting, and some uneven lighting
CTR [32] Web	14,589	Regular Scene Text	Chinese	Natural sunlight
CTR [32] Document	50,000	Regular Scene Text	Chinese	Natural sunlight, indoor lighting
CTR [32] Handwriting	23,389	Irregular scene text	Chinese	Natural sunlight, indoor lighting
IC2017-MLT [38]	1200	Irregular scene text	Multilingualism	Variations in lighting, including sunlight, indoor lighting, and shadows
IC2019-ArT [39]	4573	Irregular scene text	Chinese + English	Natural and artificial light sources
COCO-TEXT [37]	7026	Irregular scene text	English	Various lighting conditions, including daytime, dusk, and nighttime
CTW1500 [40]	2000	Irregular scene text	Chinese + English	Strong light, reflections, shadows, and nighttime scenes
MSRA-TD500 [41]	1500	Regular Scene Text	Chinese + English	Daytime and nighttime
VBC	2613	Irregular scene text	Chinese	Scenes captured under different natural lighting conditions

**Table 4:** Comparison of our method with other advanced models on the VBC and English datasets. The evaluation metrics are accuracy and average accuracy. Bold fonts indicate the best performance, while “_” denotes the second-best performance.

Method	Encoder	Input Size	VBC	ICDAR2013	SVT	ICDAR2015	SVTP	Average Accuracy (%)
RobustScanner [42]	ResNet	48 × 160	85.1	94.8	88.1	77.1	79.5	84.9
CRNN [10]	ResNet+BiLSTM	32 × 100	83.3	91.1	81.6	69.4	70.0	79.1
SVTR-T [43]	SVTR-Tiny	32 × 100	87.0	96.3	91.6	84.1	85.4	88.9
CPPD [1]	SVTR-Base	32 × 100	88.4	96.8	95.2	87.2	90.2	91.6
PPOCR [44]	ResNet	32 × 100	87.1	95.5	91.5	84.4	89.5	89.6
PARSeq [2]	ViT	32 × 128	88.0	97.0	93.6	86.5	88.9	90.8
SRN [23]	ResNet+FPN	64 × 256	86.8	95.5	91.5	82.7	85.1	88.3
CDistNet [22]	ResNet+En	32 × 128	88.1	97.4	93.5	86.0	88.7	90.7
I2C2W [3]	ResNet+En	64 × 600	86.7	95.0	91.7	82.8	83.1	87.9
LPV-B [4]	SVTR-Base	48 × 160	88.6	97.6	94.6	87.5	90.9	91.8
Ours1	ResNet	32 × 128	89.8	97.4	96.0	87.1	92.1	92.5
Ours2	PlainMamba	32 × 128	**90.8**	**98.3**	**97.1**	**89.5**	**93.4**	**93.8**

**Table 5:** Efficiency comparison across different models. Bold fonts indicate the best performance, while “_” denotes the second-best performance.

Method	Encoder	Average Accuracy (%)	Parameters (×10^6^)	FPS
RobustScanner [42]	ResNet	84.9	48.0	16.4
CRNN [10]	ResNet+BiLSTM	79.1	8.30	159
SVTR-T [43]	SVTR-Tiny	88.9	**6.03**	**408**
CPPD [1]	SVTR-Base	91.6	24.6	212
PPOCR [44]	ResNet	89.6	8.6	-
PARSeq [2]	ViT	90.8	23.8	84.7
SRN [23]	ResNet+FPN	88.3	54.7	39.4
CDistNet [22]	ResNet+En	90.7	65.5	14.5
I2C2W [3]	ResNet+En	87.9	-	-
LPV-B [4]	SVTR-Base	91.8	35.1	103
Ours1	ResNet	92.5	13.2	154
Ours2	PlainMamba	**93.8**	17.6	136

**Table 6:** Comparison of our model with others on the VBC and public datasets with metrics from [45]. The evaluation metrics are CER and WER, where smaller values indicate better performance. Bold fonts indicate the best performance, while “_” denotes the second-best performance.

Methods	IC2017-MLT		IC2019-ArT		COCO-TEXT		CTW1500		MSRA-TD500	
	CER	WER	CER	WER	CER	WER	CER	WER	CER	WER
Wang [46]	0.11	0.14	0.09	0.18	0.18	0.13	0.11	0.14	0.05	0.18
Qiao [47]	0.18	0.12	0.17	0.18	0.12	0.11	0.18	0.12	0.17	0.18
ABCNet [48]	0.17	0.12	0.17	0.13	0.12	0.11	0.17	0.12	0.17	0.13
PAN++ [49]	0.14	0.06	0.17	0.09	0.14	0.08	0.14	0.16	0.17	0.17
Ours	**0.03**	**0.06**	**0.07**	**0.05**	**0.09**	**0.07**	**0.03**	**0.05**	**0.04**	**0.06**

**Table 7:** Compares our method with SOTA methods with the results of the comparison methods obtained from [32]. Bold fonts indicate the best performance, while “_” denotes the second-best performance.

Method	Venue	Scene	Web	Document	Handwriting	Average Accuracy (%)
CRNN [10]	TPAMI2017	54.9	56.2	97.4	48.0	64.1
ASTER [17]	TPAMI2019	59.4	57.8	91.6	45.9	63.7
MORAN [50]	PR2019	54.7	49.6	91.7	30.2	56.6
SAR [16]	AAAI2019	53.8	50.5	96.2	31.0	57.9
SEED [51]	CVPR2020	45.4	31.4	96.1	21.1	48.5
MASTER [52]	PR2021	62.1	53.4	82.7	18.5	54.2
ABINet [24]	CVPR2021	60.9	51.1	91.7	13.8	54.4
TransOCR [53]	CVPR2021	67.8	62.7	97.9	51.7	70.0
CCR-CLIP [54]	ICCV2023	71.3	69.2	98.3	60.3	74.8
CPPD [1]	CVPR2023	78.4	79.3	98.9	57.6	78.6
CAM-Base [55]	CVPR2024	78.0	69.8	98.3	61.1	76.8
Ours1	-	79.2	78.5	99.1	60.7	79.4
Ours2	-	**80.7**	**80.3**	**99.5**	**61.4**	**80.5**

**Table 8:** Comparison of results using three different encoders on the VBC dataset. In all cases, DASM is used in the feature extraction stage, although its position may vary. Bold fonts indicate the best performance.

Encoder	Accuracy (%)	Parameters (×10^6^)	FPS
Ours-ResNet	88.6	**11.8**	**163**
Ours-ResNet-DASM	89.8	13.2	154
Ours-SVTR-Base	89.1	21.4	132
Ours-SVTR-Base-DASM	90.0	23.0	128
Ours-PlainMamba	90.3	16.8	152
Ours-PlainMamba-DASM	**90.8**	17.6	136

**Table 9:** Recognition accuracy under different lighting conditions. Bold fonts indicate the best performance.

Different Light Conditions	VBC
Strong light	84.4
Weak light	82.7
Lamplight	**89.5**
Darkness	73.2

**Table 10:** Comparison of our model with other models on Chinese–English mixed datasets. The performance metrics of other models are from [45] and the evaluation metric used is accuracy. Bold fonts indicate the best performance, while “_” denotes the second-best performance.

Methods	IC2019-ArT	CTW1500	MSRA-TD500	Average Accuracy (%)
Wang [46]	87.5	86.2	86.5	86.7
Qiao [47]	83.3	84.0	82.3	83.2
ABCNet [48]	81.4	81.2	80.4	81.0
PAN++ [49]	89.6	89.4	89.6	89.5
Ours	**92.4**	**92.7**	**93.2**	**92.8**

**Table 11:** Presents the ablation study of the individual units of the model. Bold fonts indicate the best performance.

Dual Attention Serial Module (DASM)	Discriminative Standard Text Font (DSTF) Module	Feature Alignment and Complementary Fusion (FACF) Module	PlainMamba	VBC	ICDAR2015	SVTP
				87.1	84.4	89.5
			✔	88.4	86.5	91.1
✔				88.0	84.8	90.2
✔			✔	88.9	86.7	91.8
	✔	✔		88.6	85.3	90.9
✔	✔	✔		89.5	86.9	92.1
	✔	✔	✔	90.2	88.1	92.9
✔	✔	✔	✔	**90.8**	**89.5**	**93.4**

## Data Availability

In this study, we collected and created the VBC Chinese dataset for natural scenes, which includes text images captured under various conditions such as strong light, low light, darkness, and artificial lighting. This dataset is freely available for researchers to use, and it can be accessed at the following URL: https://1264sw/VBC (github.com). Accessed on 11 October 2024.
